# Sigma hole donor XB and ChB catalysis in oligosaccharide synthesis

**DOI:** 10.1016/j.trechm.2025.07.001

**Published:** 2025-10

**Authors:** Mukul Mahanti, M. Carmen Galan

**Affiliations:** 1 School of Chemistry, Cantock’s Close, University of Bristol, BS8 4PU, Bristol, UK

## Abstract

Sigma hole-based noncovalent interactions (NCIs), such as halogen bonding (XB) and chalcogen bonding (ChB), offer a powerful alternative to hydrogen bonding (HB) in stereoselective catalysis. The high directionality and tunability of these catalysts have been harnessed to facilitate more efficient glycosylation processes under mild conditions. Recent advances highlight the significant yet underexplored potential of these interactions, paving the way for novel and improved catalytic strategies in carbohydrate chemistry with unique activation modes.

## Background

The use of NCIs in catalysis to achieve high stereoselective and efficient transformations has gained significant attention in recent years. The structural design of these organocatalysts can influence substrate recognition, stabilize transition states, and enhance overall reaction efficiency through the precise manipulation of energetic and stereochemical features of intermolecular forces. Traditionally, HB has dominated the field due to its strength and directionality, enabling highly asymmetric transformations. However, HB interactions, classified as ‘hard’ Lewis acids, often show limited effectiveness with softer substrates, restricting their applicability in complex molecular settings.

To address these limitations, sigma hole-based interactions, such as XB and ChB, have emerged as promising alternatives [1]. Unlike HB, these interactions involve electropositive regions formed due to anisotropic electron density distributions along the R–X or R–Ch bond, allowing halogens and chalcogens to adopt a Lewis acidic role that enables strong, directional, and tunable NCIs with Lewis basic substrates, making these reagents valuable tools for catalysis. It is well-established, and widely recognized in crystal engineering and supramolecular chemistry, that these XB and ChB catalysts interact through sigma hole interactions; their applications in solution-phase catalysis, particularly in stereoselective carbohydrate synthesis are now emerging [2].

The application of sigma hole-based NCIs in glycosylation chemistry has recently gained significant momentum due to their ability to facilitate challenging bond-forming processes with high stereocontrol and regioselectivity under mild conditions. This is achieved by mimicking enzyme-like substrate recognition, eliminating the need for harsh promoters or restrictive protecting group strategies. In this focus article, we will highlight pioneering studies of XB and ChB catalysis in oligosaccharide synthesis that operate via non-protic activation mechanisms. These strategies enable glycosylation under milder yet more robust conditions compared with traditional approaches.

### XB-catalyzed glycosylations

**Figure 1:**
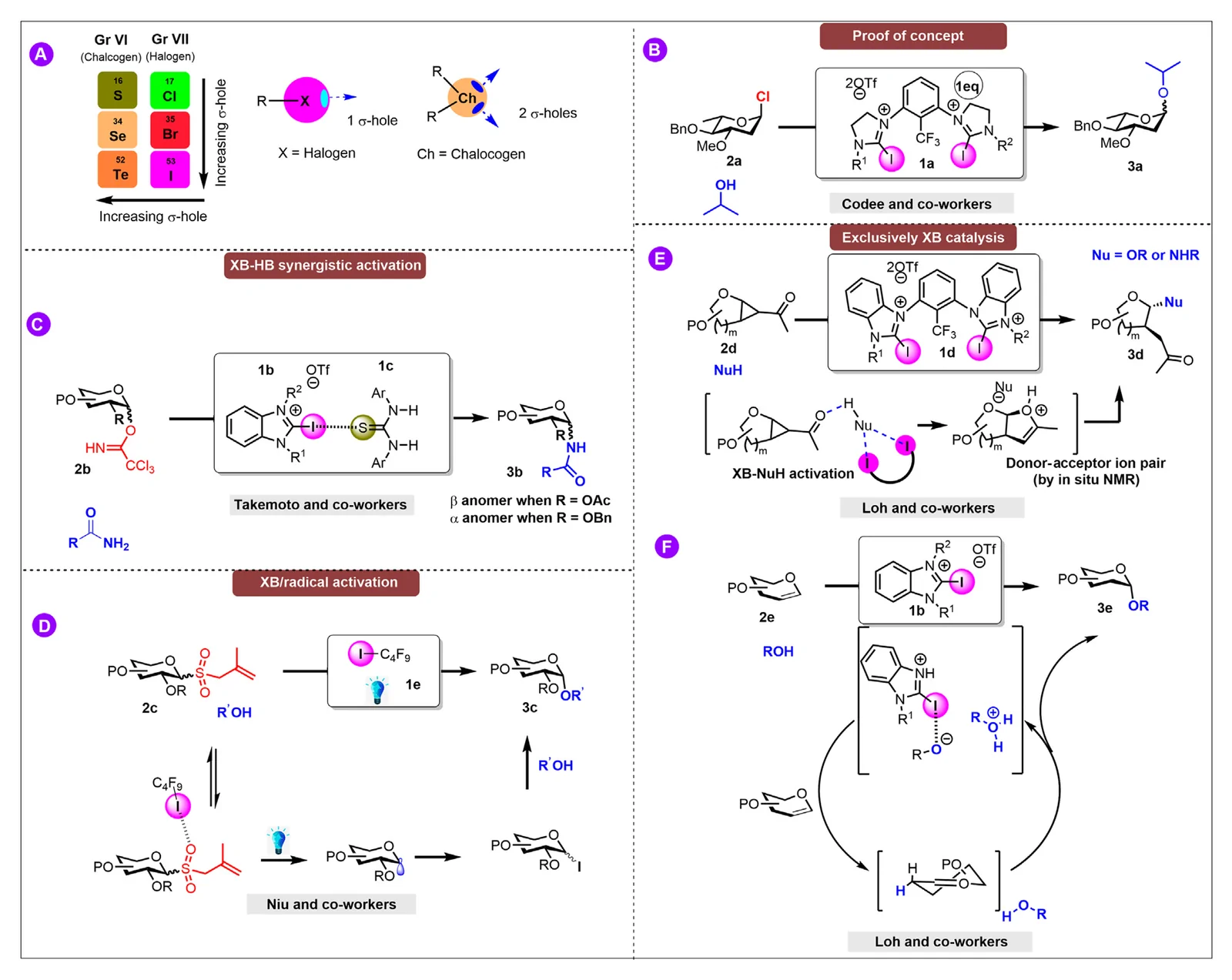
Examples of XB-catalysis applied to glycosylation chemistry. Abbreviations: HB, hydrogen bonding; XB, halogen bonding. See [3,4,6–8].

Although one of the first examples of pyranosyl halide activation using an stoichiometric XB promoter **1a** in a Koenigs−Knorr-type glycosylation was reported by Codee, Huber, and coworkers (Figure 1B) [3], Takemoto and coworkers [4] documented one of the early examples of XB-thiourea co-catalyzed glyco-functionalization in 2018. The team disclosed a synergistic activation approach based on 2-iodoazolium salts (**1b**) and thiourea (**1c**) (XB-HB), where XB (2-iodoazonium salts) enhanced the acidity of thiourea and thus activated the glycosyl trichloroacetmidates **2b** for the formation of *β*-*N*-glycosylamides from amides, a challenging substrate due to its poor nucleophilicity (Figure 1C). It was envisioned that a soft and mild Lewis acid such as 2-iodoazolium salt (**1b**), an XB donor, could act as a co-catalyst and interact with the soft Lewis basic thiourea (**1c**) to increase its HB-donor ability towards anomeric trichloroacetimidate activation [4]. Later on, the team demonstrated this type of synergistic activation can also be used to access *α*-*N*-glycosides by replacing the benzoyl/acetyl group at the C2 position with a benzyl group [5].

Further, Niu and coworkers introduced a novel XB-assisted radical-based stereoconvergent 1,2-*cis*-glycosylation strategy involving allyl glycosyl sulfones (**2c**) (Figure 1D) [6]. The reaction proceeded via a halogen-bond (XB) complex between the sulfone donor and C_4_F_9_I. Upon visible light irradiation, this complex underwent a single-electron transfer cascade, generating a glycosyl radical, which then formed a reactive glycosyl iodide intermediate. This electrophilic species can subsequently react with a nucleophile, forming glycosidic bonds with high stereoselectivity. The method avoided the use of harsh reagents, metal catalysts, or strong acids, offering a milder alternative for constructing challenging 1,2-cis-glycosides. Furthermore, computational studies were used to validate the key role of XB in facilitating electron transfer.

In 2019, Loh and coworkers reported the first example of on exclusively XB-catalyzed glycosylation [7]. Unlike previous approaches that relied on HB or Brønsted acid catalysis, this work established XB-catalysis as a standalone mode of activation, demonstrating its efficiency in achieving anomeric-selective and stereospecific *O-* and *N*-glycosylation under mild conditions (Figure 1E). The study focused on a strain-release glycosylation strategy using (D)-furanoxylose-derived glycosyl donors (**2d**) in which the XB catalyst (**1d**) concomitantly activated both the donor and the nucleophile or acceptor via an unprecedented mechanism, which entailed a multistage catalytic cycle involving donor–acceptor ion-pair species as key intermediates. Control experiments confirmed that in contrast to conventional Brønsted acid or HB-catalysis, XB-catalysis remains operative throughout all stages of the reaction.

Building upon this foundational work, the Loh team expanded the use of XB-catalysis (**1b**) to glycal (**2e**) activation (Figure 1F) [8], demonstrating its potential in the *α*-stereoselective synthesis of 2-deoxyglycosides, a class of biologically important natural products that are traditionally challenging to access [9]. Halide competition assays, deuterium-labeling studies, and kinetic isotope effect (KIE) analysis reveal a stepwise mechanism whereby the XB-catalyst facilitates proton transfer from the OH nucleophile to the glycal C2. Large primary KIEs suggested quantum tunneling in the rate-determining step. Additionally, *in situ* NMR studies uncovered a sigmoidal kinetic profile, indicative of slow formation of an XB-activated intermediate, further differentiating the reaction from traditional Brønsted acid catalysis. These studies highlight the catalytic potential of XB-catalysis over HB-catalyzed protocols, which tend to be milder and thus require higher temperatures or co-catalysts to be efficient [10,11].

### ChB-catalyzed glycosylation

**Figure 2:**
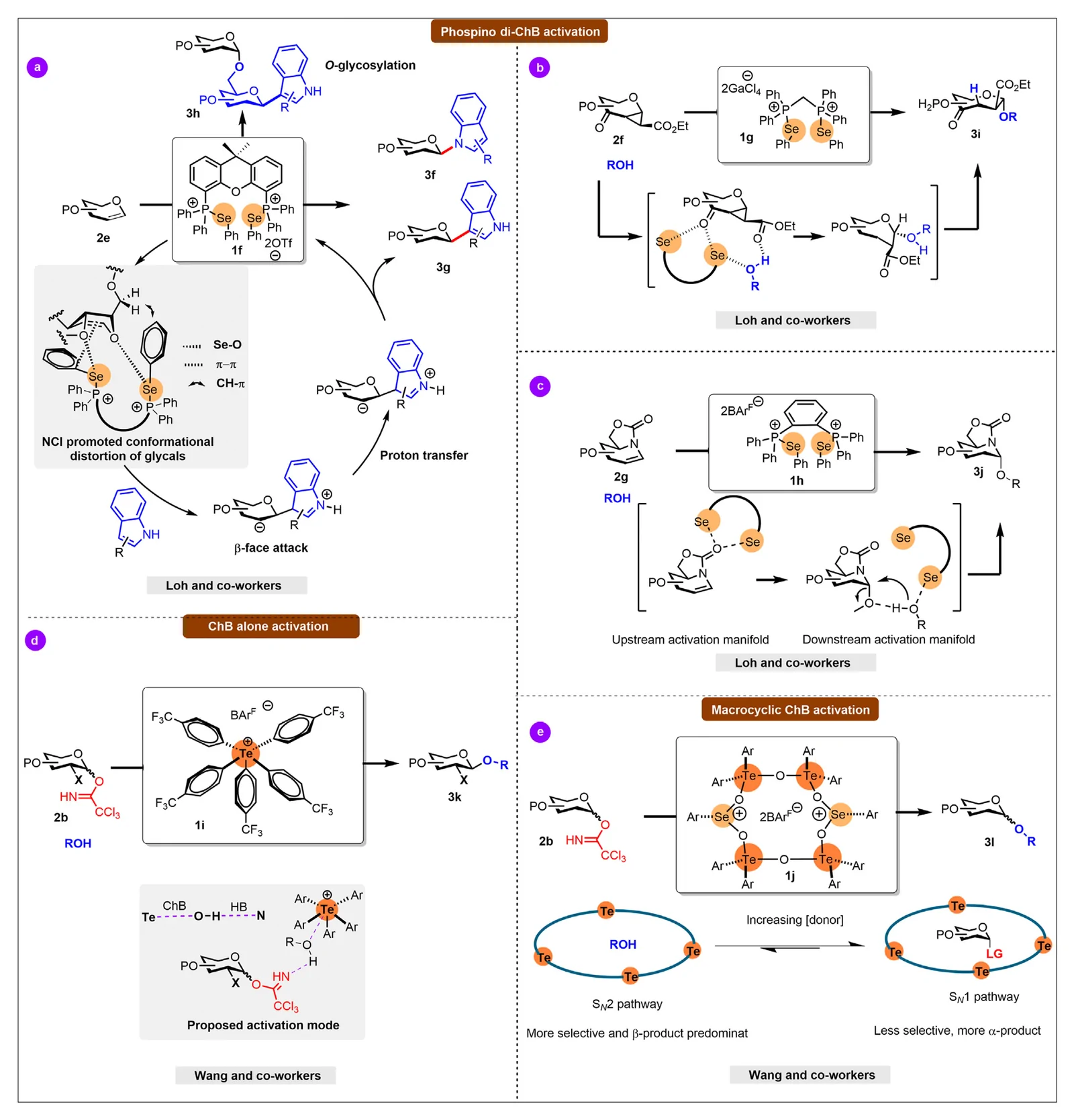
Examples of ChB catalysis applied to glycosylation chemistry. Abbreviations: ChB, chalcogen bonding; NCl, noncovalent interaction. See [7,8,12].

In contrast to halogen atoms in XB-catalysis (Figure 1A), each chalcogenide atom is capable of establishing two sigma holes. This distinctive feature endows ChB-catalysis with unique activation pathways and enhanced directionality that are inaccessible through XB-systems. Motivated by this concept and inspired by the pioneering work of Wang and coworkers [12], where they showed phosphonium chalcogenide (PCH) can facilitate C–H activation through Ch–π interaction, Loh and coworkers demonstrated that the same class of catalysts, diphosphine-diselenium (P_2_Se_2_), (**1f**) could also activate glycals to facilitate *β*-selectivity to form *N-* and *C*-glycosides (**3f**, **3g**) (Figure 2A) [13]. They proposed that the catalyst interaction underpins the change in conformation in the glycal to form the key reactive intermediate, thus favoring delivery of the indole nucleophile from the *β*-face. NMR and computational studies confirmed the role of Se–O and π-based interactions in stabilizing the reactive intermediates, leading to the high selectivity. Additionally, this catalyst was also able to produce 2-deoxy *O*-glycosides (**3h**) from glycals but gave α-selectivity, instead. In an another study, this class of catalyst has been exemplified in a strain-release glycosylation to synthesize seven-membered sugars (Figure 2B) [14]. It was shown in the mechanistic outlook of the catalyst that the selenium centers provided bifurcated ChB for enhanced NCI-activation and stereocontrol, while phosphine units contribute pnictogen bonding (PnB) to stabilize the reaction intermediates (**3i**). Mechanistically, the catalyst stabilizes the glycosyl acceptor’s hydroxyl group via ChB and HB, while ChB interactions with the donor’s carbonyl groups form a noncovalent ternary complex. This preorganized assembly fixes the glycosyl acceptor on the *α*-face, ensuring controlled aglycone delivery. Another variant of these type of diphosphine-diselenium catalysts (**1h**) was also described by Loh and coworkers for iminoglycosylation of bicyclic iminoglycal donor (**2g**) to generate glycomimetics of therapeutic importance (Figure 2C) [15]. Mechanistic investigations revealed multiple concurrent-ChB interactions with the donor alone and donor–acceptor, respectively, in the upstream and downstream manifold, which led to the high selectivity. More recently, the Wang group introduced tellurium chalcogen as a novel glycosylation catalyst (Figure 2D). Hexavalent tellurium (**1i**) was used to activate trichloroacetitmidate donors (**2b**) to produce *β*-glycosides in glycosylations with diol-containing glycosides [16]. Te(VI) featured a confined interaction site that concurrently selectively engaged the hydroxyl group in the glycosyl acceptor and the leaving group of the donor, favoring *β*-(1,2)-linkage formation, in the absence of C-2 neighboring participating groups, whilst suppressing the formation of alternative regio-isomers. It is also proposed that the Te(VI) catalyst stabilized the oxocarbenium intermediate and restricted the axial nucleophile approach, which could explain the stereoselectivity observed. In a very recent study, Wang and coworkers introduced the first example of a macrocyclic ChB for selective glycosylation, which they call the ‘dynamic glycosylation approach’. In their study they used tellurium-based macrocycle (**1j**) to control the glycosylation [17]. They proposed that the catalyst formed dynamic host–guest complexes with glycosyl donors or acceptors, enabling a tunable shift between S_*N*_1 and S_*N*_2 mechanisms based on reactant concentration. This dynamic process allowed precise stereoselective control, favoring *β*-selectivity under higher acceptor concentrations through an S_*N*_2 pathway, whereas at higher donor concentrations, an S_*N*_1 pathway and α-selectivity was favored.

### Concluding remarks

Sigma hole-based NCIs, particularly XB and ChB, have revolutionized glycosylation strategies by offering efficient alternatives to traditional HB or Brønsted acid catalysis. The exploitation of NCIs in catalysis has shown enhanced reaction efficiency and stereoselectivity under mild conditions and with novel modes of activation, allowing a wider substrate scope and efficient access to complex carbohydrate structures with precise anomeric control. Future developments should focus on expanding the substrate scope, enhancing catalyst design, and furthering our understanding of reaction pathways, so that improved catalytic systems can be developed for every glycosidic linkage, perhaps by integrating with other complementary catalysis modes. Access to molecular synthons, not previously accessible, will allow the exploration of applications in drug discovery by, for example, developing glycomimetics. The development of more efficient and sustainable reagents will be pivotal for advancing glycosylation chemistry in the pharmaceutical and industrial contexts.
